# The comparison between anterior and posterior approaches for removal of infected lumbar interbody cages and a proposal regarding the use of endoscope-assisted technique

**DOI:** 10.1186/s13018-021-02535-x

**Published:** 2021-06-16

**Authors:** Yun-Da Li, Jia-En Chi, Ping-Yeh Chiu, Fu-Cheng Kao, Po-Liang Lai, Tsung-Ting Tsai

**Affiliations:** 1 Department of Orthopedic Surgery, Spine Section, Bone and Joint Research Center, Chang Gung Memorial Hospital and Chang Gung University College of Medicine, Taoyuan, Taiwan; 2 Department of Orthopedic Surgery, New Taipei Municipal TuCheng Hospital (Built and Operated by Chang Gung Medical Foundation), New Taipei City, Taiwan

**Keywords:** Cage infection, Lumbar interbody fusion, Surgical approach, Cage removal, Endoscope

## Abstract

**Background:**

In cases of postoperative deep wound infection after interbody fusion with cages, it is often difficult to decide whether to preserve or remove the cages, and there is no consensus on the optimal approach for removing cages. The aim of this study was to investigate the surgical management of cage infection after lumbar interbody fusion.

**Methods:**

A retrospective study was conducted between January 2012 and August 2018. Patients were included if they had postoperative deep wound infection and required cage removal. Clinical outcomes, including operative parameters, visual analog scale, neurologic status, and fusion status, were assessed and compared between anterior and posterior approaches for cage removal.

**Results:**

Of 130 patients who developed postoperative infection and required surgical debridement, 25 (27 levels) were diagnosed with cage infection. Twelve underwent an anterior approach, while 13 underwent cage removal with a posterior approach. Significant differences were observed between the anterior and posterior approaches in elapsed time to the diagnosis of cage infection, operative time, and hospital stay. All patients had better or stationary American Spinal Injury Association impairment scale, but one case of recurrence in adjacent disc 3 months after the surgery.

**Conclusions:**

Both anterior and posterior approaches for cage removal, followed by interbody debridement and fusion with bone grafts, were feasible methods and offered promising results. An anterior approach often requires an additional extension of posterior instrumentation due to the high incidence of concurrent pedicle screw loosening. The use of an endoscope-assisted technique is suggested to facilitate safe removal of cages.

## Introduction

Because of rising life expectancy, the demand for spinal surgery among elderly individuals has increased, and lumbar spinal fusion has become one of the most commonly performed spinal surgery procedures [1, 2]. Interbody fusion, either by posterior lumbar interbody fusion (PLIF) or transforaminal lumbar interbody fusion (TLIF), has better clinical outcomes than posterior fusion or posterolateral fusion (PLF) for the treatment of degenerative spondylolisthesis [3, 4]. In addition, interbody fusion with cage has several advantages, including the restoration of intervertebral disc height and foraminal dimensions and the maintenance of lumbar lordosis without the need to harvest autologous structural bone graft [5].

Although interbody cages can produce satisfactory results, they are associated with several complications, including subsidence, migration/dislodgement, pseudarthrosis, and infection [6, 7]. The reported incidence of deep wound infection after lumbar interbody fusion was 1.3–7.2% [8, 9]. Because an interbody cage is a foreign body and a communicating tract is created at the time of cage insertion, the use of such a cage always entails some risk of deep wound infection extending into or from the cage space. In a previous study, 62.5% patients treated with posterior lumbar interbody fusion (PLIF) who had a postoperative infection were found to have infectious spondylitis around the interbody cages and grafted bone [10]. In the management of deep wound infection after instrumentation and interbody fusion, it is often difficult to decide whether to preserve or remove the cage, because the removal of a cage is technically demanding, associated with a high risk of dural and root injury, and may result in spinal instability. There is also no consensus about the optimal approach for the removal of an infected cage and very limited data available regarding management efforts in such situations. Therefore, the aims of this study were to investigate the appropriate approach for the removal of infected cages and the outcomes of surgeries aimed at treating postoperative interbody cage infection in the lumbar spine.

## Materials and methods

### Data collection

A retrospective review of patients treated between January 2012 and August 2018 was conducted. Patients were included for statistical analysis if they (1) were aged ≥ 18 years at the time of surgery; (2) underwent lumbar spine interbody fusion with cages via anterior lumbar interbody fusion (ALIF), transforaminal lumbar interbody fusion (TLIF), or posterior lumbar interbody fusion (PLIF); (3) had surgical debridement due to postoperative wound infection; and (4) required removal of a cage due to the impression of cage infection. This study was approved by the Institutional Review Board of Chang Gung Medical Foundation (IRB No. 201900512B0). The IRB reviewed and determined that it is expedited review according to Case research or cases treated or diagnosed by clinical routines, with the approval for the waiver of informed consent.

In our principles of treatment, the indications of surgical debridement for postoperative wound infection included the failure of conservative treatment, poor wound healing, progressive neurologic deficits, significant vertebral instability, intractable pain, and the need for specimen collection. Because an interbody cage is a foreign body, the retention of an infected cage may obstruct infection control. A loose cage also entails risks of migration to the epidural or retroperitoneal space, which may result in the compromise of the canal, nerve roots, and vascular structures. Therefore, the removal of a cage was indicated if the impression of a cage infection was indicated preoperatively or intraoperatively. Cage infection was diagnosed based on the following conditions: (1) the patient developed progressive signs and symptoms, such as fever, back pain, and sciatica postoperatively; (2) follow-up radiographic images showed vertebral end plate destruction with cage subsidence or cage migration, and follow-up magnetic resonance imaging (MRI) revealed signal changes of abutting vertebrae in T1-weighted images or obvious accumulation of excessive fluid around the cage space in T2-weighted images; (3) laboratory data revealed leukocytosis, as well as elevated C-reactive protein (CRP) and erythrocyte sedimentation rate (ESR); (4) intraoperative findings revealed a sinus tract at the entrance of cage space; or (5) uncontrollable infection even with repeated surgical debridement with retention of the unmoved cage. The first three conditions are often considered as the necessary criteria for the diagnosis of cage infection. All authors listed in this article performed the surgeries or were involved with the index treatment of the enrolled patients.

Preoperative patient characteristics including age, gender, visual analogue scale (VAS) score, neurologic status, findings of radiographic studies and MRI, the degree of end-plate destruction, spinal levels and methods of initial surgery, and elapsed time to a diagnosis of cage infection were recorded. Perioperative details of debridement were investigated, including the surgical approach, stability of previous transpedicular screws, blood loss, operative time, methods for removal of cages, and whether bone graft was used for interbody fusion. In addition, postoperative data, including tissue culture results, the duration of intravenous antibiotics administration and hospital stay, recurrence rates within the follow-up period, postoperative 12-month VAS score, postoperative 12-month neurologic status, and the interbody fusion status, were also reviewed.

Recurrence was defined as having occurred if a patient had recurrent symptoms and signs such as back pain, fever, leukocytosis, and elevated CRP and ESR after a previous complete course of treatment and needed to undergo antibiotic treatment or unplanned surgery within 12 months. The interbody fusion status was evaluated by plain films or computed tomography (CT) scans taken 12 months after the surgery. Solid union was defined as continuous trabecular bridging across the interface of the treated segments, whereas the presence of radiolucent zone at the treated segments was considered to indicate pseudarthrosis. Between these two statuses, partial union was indicated.

### Operative procedures and endoscope-assisted removal technique

When cage removal was decided upon preoperatively, comprehensive surgical planning was required. The surgical approaches that could be used included anterior, posterior, or combined anterior + posterior procedures on the same day or staged procedures. The surgical approach was determined for each patient individually according to his or her comorbidities, the elapsed time to the diagnosis of cage infection, the extent of infection, the location of the cage, the severity of bony destruction, the stability of instrumentation, and the preference of the surgeon.

For an anterior approach, the patient was placed in the lateral decubitus position with the major infected side up. The procedure was started with the flank retroperitoneal approach, which was followed by debridement, removal of the cage, and ALIF with bone grafts. The bone grafts may be autologous tricortical iliac bone graft or structural allograft. Lateral instrumentation with screws may be applied according to stability and individual surgical planning. A posterior approach was performed with the patient placed on a four-poster spinal frame in the prone position. One posterior midline skin incision was made, followed by infection eradication around the epidural and posterolateral space. After the entrance of the previous TLIF/PLIF tract was identified, a shaver or paddle distractor could be used to enlarge the entrance and tract. Then, an attempt to retrieve the cage was made, followed by transforaminal lumbar interbody debridement and fusion (TLIDF) with bone grafts, which could be autologous iliac bone graft or morselized allograft. If there was difficulty in removal through the previous tract, the tract could be prepared on the contralateral side in order to enlarge the intervertebral height, which facilitated the retrieval of the cage from either the ipsilateral or contralateral tract. The level of the upper instrumented vertebra (UIV) and the lowest instrumented vertebra (LIV) may need to be extended due to the high probability of simultaneous screw loosening. The extension of the instrumented levels was indicated if there’s evidence of pedicle screw loosening, which was determined by the halo sign in preoperative radiographic images or intraoperative finding.

**Fig. 1:**
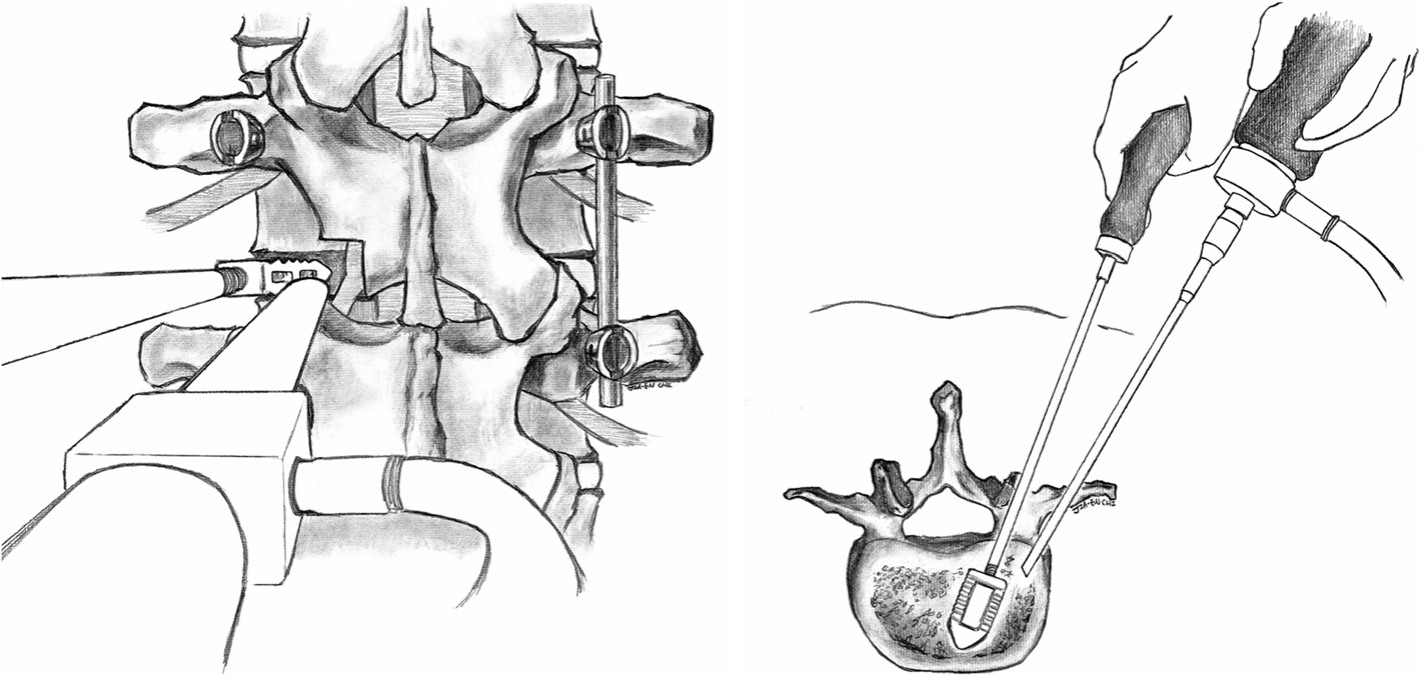
Illustrations of posterior and axial view, respectively, of the endoscope-assisted removal technique (**A**, **B**). By means of adequate enlargement of the previous TLIF/PLIF entrance, the endoscope and working instruments can be inserted concurrently to identify the cage and adjust the cage alignment safely and efficiently. After thread tightening, the cage can then be retrieved smoothly

**Fig. 2:**
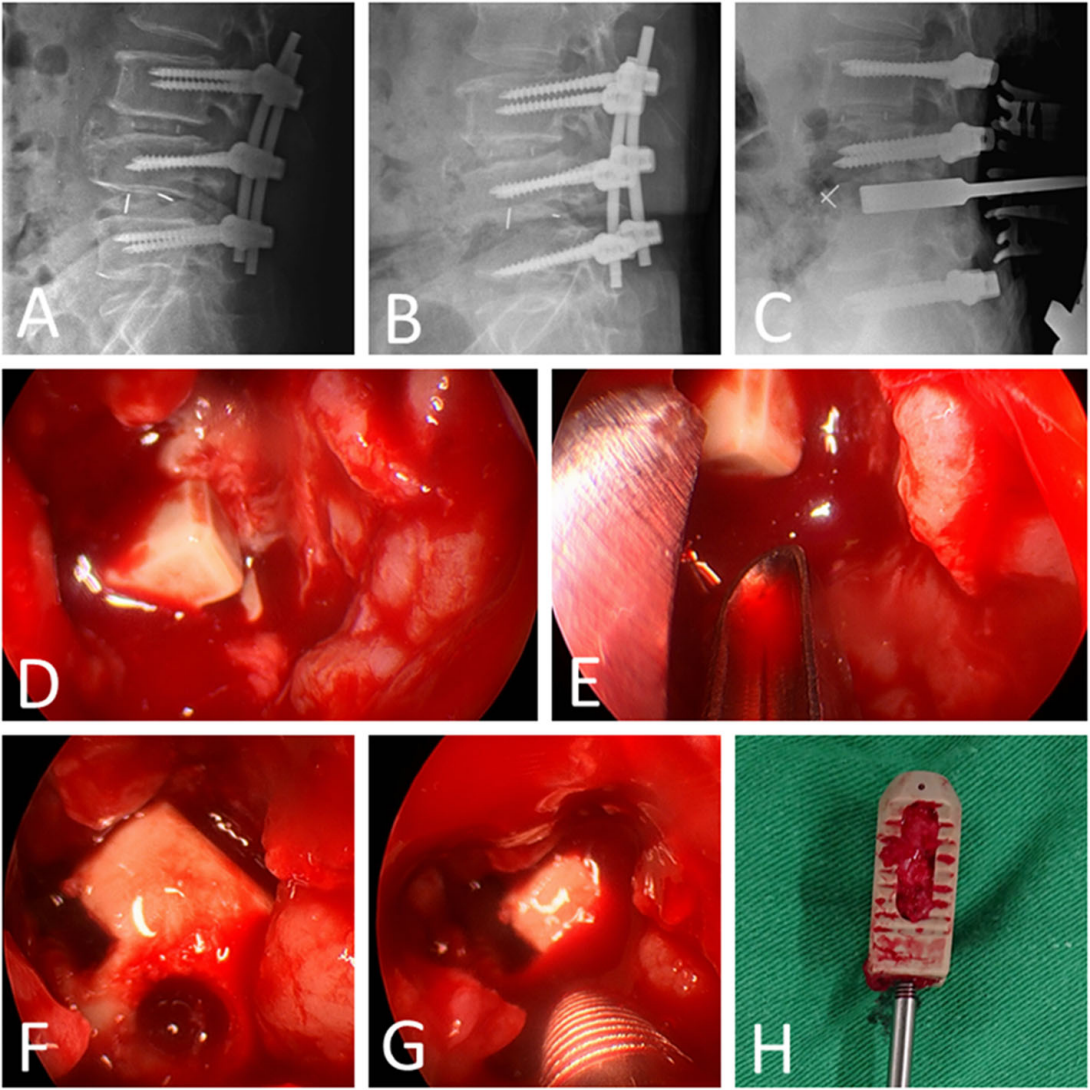
**A** A 54-year-old man underwent L3–L5 fusion with instrumentation and cages, and the initial postoperative X-ray showed good screw and cage positions. **B** One month later, the patient developed severe back pain, leukocytosis, and elevated CRP level. An X-ray revealed end plate destruction at L4–L5, posterior migration of the L4–L5 cage, and L5 screw loosening. **C** A posterior approach was chosen, and an intraoperative X-ray showed that the cage was pushed to the anterior region during the process of entrance enlargement with the TLIF shaver. **D** With an endoscopy-assisted technique, the cage was identified clearly. **E** A disk clamp was used to adjust the axis of the cage. **F** The axis of the cage was adjusted to let the connector hole for the threaded cage holder be directed along with the trajectory of the tract. **F**, **H** Thread tightening could be performed under a clear visual filed, and then the cage was retrieved

Because the entrance diameter of the previous TLIF/PLIF tract was typically small, often less than 10 mm, it was difficult to find and clamp the migrated cage during the blind retrieval process from a posterior approach. Therefore, in two most recent cases, we tried to use an endoscope to facilitate the process of cage removal during posterior surgery (Fig. 1). In these two endoscope-assisted cases, the clamping of cage was initially operated without endoscope, and then endoscope was used to get better spatial positioning of the loose cage. Standard instruments for knee/shoulder arthroscopy, such as a 30° 4-mm rigid arthroscope, and an image intensifier were utilized. A dry scope technique was used due to the open surgical field. In the first step, after the entrance of the previous TLIF/PLIF tract was identified and enlarged, the endoscope was inserted through the tract. A suction tip could be put in the tract concurrently to maintain the clearness of the visual field. Then, we could identify the location and alignment of the cage. In the second step, we could adjust the axis of the cage by grasper or disk clamp under endoscopic guidance. The axis of the cage should be adjusted to let the connector hole for the threaded cage holder be directed along the trajectory of the tract. Next, the threaded cage holder can be inserted through the tract to attach the connector hole on the cage efficiently under endoscopic guidance. After thread tightening, the cage can then be retrieved smoothly (Fig. 2).

### Postoperative care

Empiric intravenous antibiotics were initiated in each patient immediately after the surgery, and then adjusted according to the culture reports and recommendations from the physicians of infectious diseases. The duration of intravenous antibiotics, usually 4–6 weeks, depended on the clinical symptoms and follow-up laboratory data (including white blood count with a differential count, ESR, and CRP level). After discharge, oral antibiotics were prescribed for 1–3 months at the outpatient department. All the patients were advised to wear a Taylor brace during ambulation for 3 months. The patients were followed up at 1, 3, and 6 months and then annually.

### Statistical analysis

Preoperative, perioperative, and postoperative data were then compared between the patients who underwent an anterior approach and those who underwent a posterior approach for the removal of cages. Categorical variables were compared using the Pearson χ2 or Fisher’s exact test. Continuous variables were compared using the Student’s t test. Statistical analysis was performed using SPSS 22.0 (IBM, Armonk, New York). The level of significance was established at a *p* value of less than 0.05.

## Results

### Patient characteristics

During the study period, a total of 6178 patients underwent spinal fusion with interbody cages (mostly TLIF and PLIF) at our institution. There were 130 cases of postoperative infection which required surgical debridement. One hundred twenty-two out of 6178 cases (2.0%) developed at the authors’ hospital and eight cases were referred from other hospitals. Among these 130 patients, 25 (who were affected at 27 levels in total) were diagnosed with cage infection and included in our study. Therefore, in addition to surgical debridement, cases of cage infection also underwent the removal of cages and interbody fusion with bone grafts through either an anterior or posterior approach. The patient characteristics are summarized in Table 1. The average age of the patients at surgery was 65 ± 8.6 years (range, 50–84 years), and there were 18 males and 7 females. The most common infected cage level was L4–L5 (14/27 = 52%), followed by L5–S1 (5/27 = 19%) and L3–L4 (4/27 = 15%). Cage migration (13/27 = 48%) and cage subsidence (12/27 = 44%) were the two major image characteristics. The Kulowski’s classification was used to evaluate the degree of end-plate destruction in infectious spondylitis (Grade I: only disk space narrowing; Grade II: bony destruction limited only in the end-plate; Grade III: vertebral body destruction of less than 50% of vertebral height; Grade IV: vertebral body destruction over 50% of vertebral height). In this study, the cases were classified from Grade II to Grade IV. The mean elapsed time to the diagnosis of cage infection from the previous primary surgery was 72.1 days (range, 27–170 days). Twelve patients underwent an anterior approach to remove the infected cage, and 13 underwent a posterior approach for removal of the cage. There were 21 patients (84%) who sustained pedicle screw loosening as determined by preoperative radiographic images or intraoperative findings and needed an extension of the instrumented level. The mean hospital stay and duration of intravenous antibiotics use after operation was 29.3 days (range, 7–70 days). The mean period of follow-up was 15.7 months (range, 12–60 months).

**Table 1:** Demographic data and outcomes of patients who underwent cage removal

	Number of cases (n = 25)	Number of levels (n = 27)
Age (years)	65.6 ± 8.6 (50–84)	
Gender Male Female	18 (72%) 7 (28%)	
Infected cage level L1–L2 L2–L3 L3–L4 L4–L5 L5–S1		3 (11%) 1 (4%) 4 (15%) 14 (52%) 5 (19%)
Image cage features Cage migration Posterior Anterior Cage subsidence Fluid accumulation around disc		13 (48%) 11 (85%) 2 (15%) 12 (44%) 2 (8%)
Elapsed time to the diagnosis (days)	72.1 (27–170)	
Approach for cage removal		
Anterior Posterior	12 (48%) 13 (52%)	
Screw loosening	21 (84%)	
Hospital stay after operation (days)	29.3 (7–70)	
Period of follow-up (months)	15.7 (12–60)	
Recurrence	1 (4%)	
Fusion status		
Solid union Partial union Pseudarthrosis	21 (84%) 3 (12%) 1 (4%)	

Data presented as mean (range), mean ± standard deviation, or n (%)

### Infection control

The intraoperative culture results are shown in Table 2. The most common pathogen was methicillin-resistant *Staphylococcus aureus* (20%), followed by coagulase-negative *Staphylococcus* (16%), *Pseudomonas aeruginosa* (8%), *Propionibacterium* species (8%), and *Candida albicans* (8%), and methicillin-sensitive *Staphylococcus aureus* (4%). Under our treatment algorithms, there was only one case of recurrence in an adjacent disc 3 months after the surgery. In that case, the revision surgery was performed with the extension of instrumented level and interbody debridement and fusion via posterior surgery, and adequate infection control and union of the interbody fusion was noted.

**Table 2:** Culture results of cage infection

Pathogens	n (%)
MRSA	5 (20)
CoNS	4 (16)
*Pseudomonas aeruginosa*	2 (8)
*Propionibacterium* species	2 (8)
*Candida albicans*	2 (8)
MSSA	1 (4)
No organism	9 (34)
Culture rate	16 (64)

*MRSA*, methicillin-resistant *Staphylococcus aureus*; *MSSA*, methicillin-sensitive *Staphylococcus aureus*; *CoNS, c*oagulase-negative *Staphylococci*

Data presented as n (%)

### Interbody fusion status and outcomes

The interbody fusion status was evaluated by plain films or CT scans taken 12 months after the surgery. Solid union, partial union, and pseudarthrosis occurred in 84% (21/25), 12% (3/25), and 4% (1/25) of the patients, respectively. The mean VAS score of back pain improved from 7.0 ± 1.4 preoperatively to 3.5 ± 1.4, 2.8 ± 1.6, and 1.4 ± 1.0 at 1 week, 1 month, and 12 months postoperatively, respectively.

### Neurologic status

The preoperative neurologic status was American Spinal Injury Association (ASIA) impairment scale C in 2 patients, ASIA D in 8 patients, and ASIA E in 15 patients. The postoperative neurologic status 12 months after surgery was ASIA C in 1 patient, ASIA D in 5 patients, and ASIA E in 19 patients. All the patients had a better or unchanged ASIA scale status. No intraoperative nerve root injury was noted for any of the cases, whether an anterior or posterior approach was used.

### Comparisons between anterior and posterior approaches

**Fig. 3:**
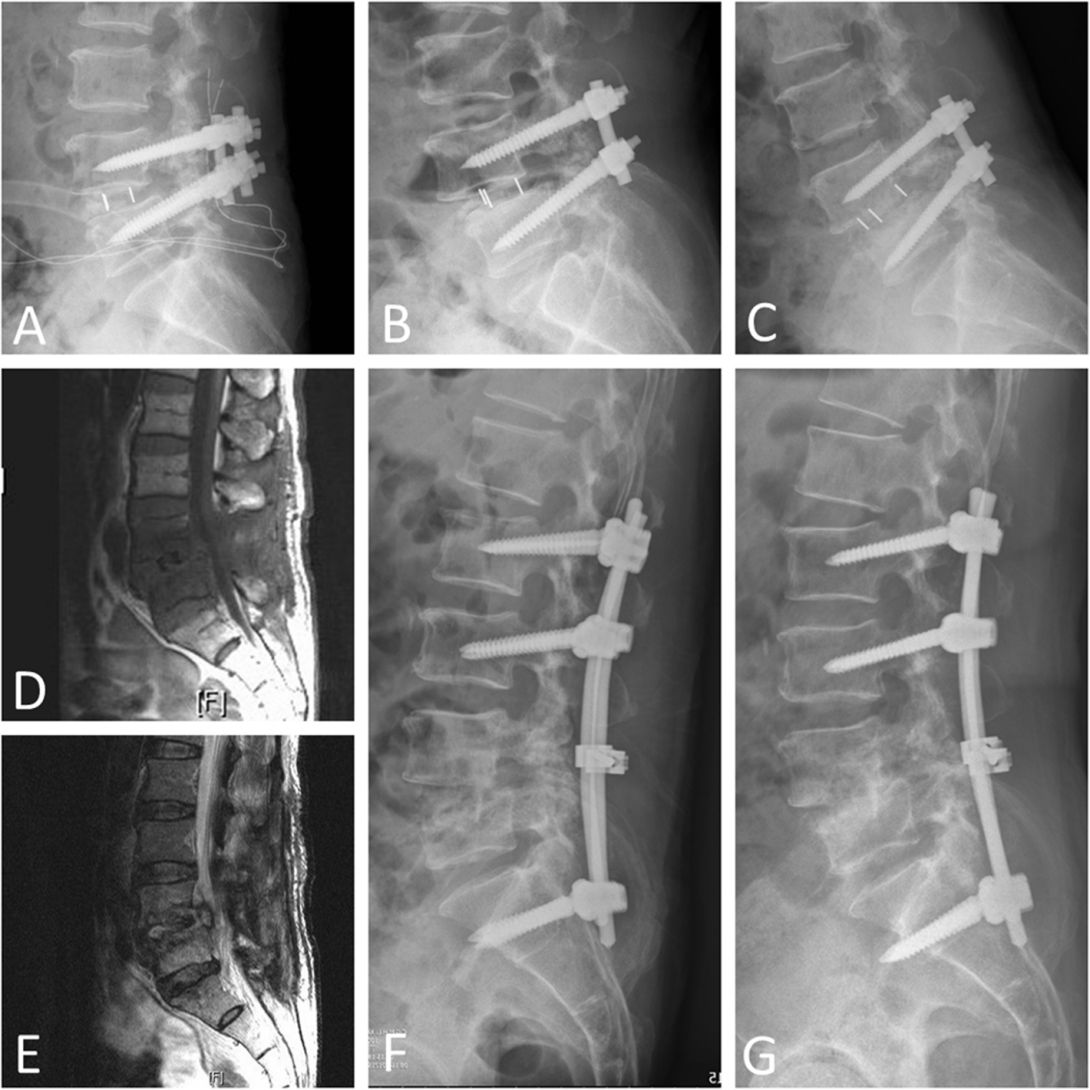
**A** A 70-year-old man underwent L4–L5 fusion with screws and cage. An initial postoperative X-ray showed good locations of the implants. **B** One month after the surgery, a follow-up X-ray showed posterior migration of the L4–L5 cage and suspected L5 screw loosening, and conservative treatment with oral analgesics and brace protection was chosen. **C** Three months after the surgery, progressive posterior migration of the L4–L5 cage, erosion of the L4–L5 end plates, and L5 screw loosening were noted. **D**, **E** T1-weighted and T2-weighted magnetic resonance imaging revealed low signal changes in the abutting L4 and L5 vertebrae, and fluid accumulation in the L4–L5 disc space, respectively. **F** An anterior + posterior approach was chosen. Posterior surgery for the upper and lower extension of the instrumented level was performed, followed by anterior surgery with cage removal and interbody fusion with autologous iliac tricortical bone graft. An initial postoperative X-ray showed good alignment. **G** Fourteen months after the anterior + posterior surgery, an X-ray revealed L4–L5 solid interbody union. The patient also had improved clinical outcomes and adequate infection control.

**Fig. 4:**
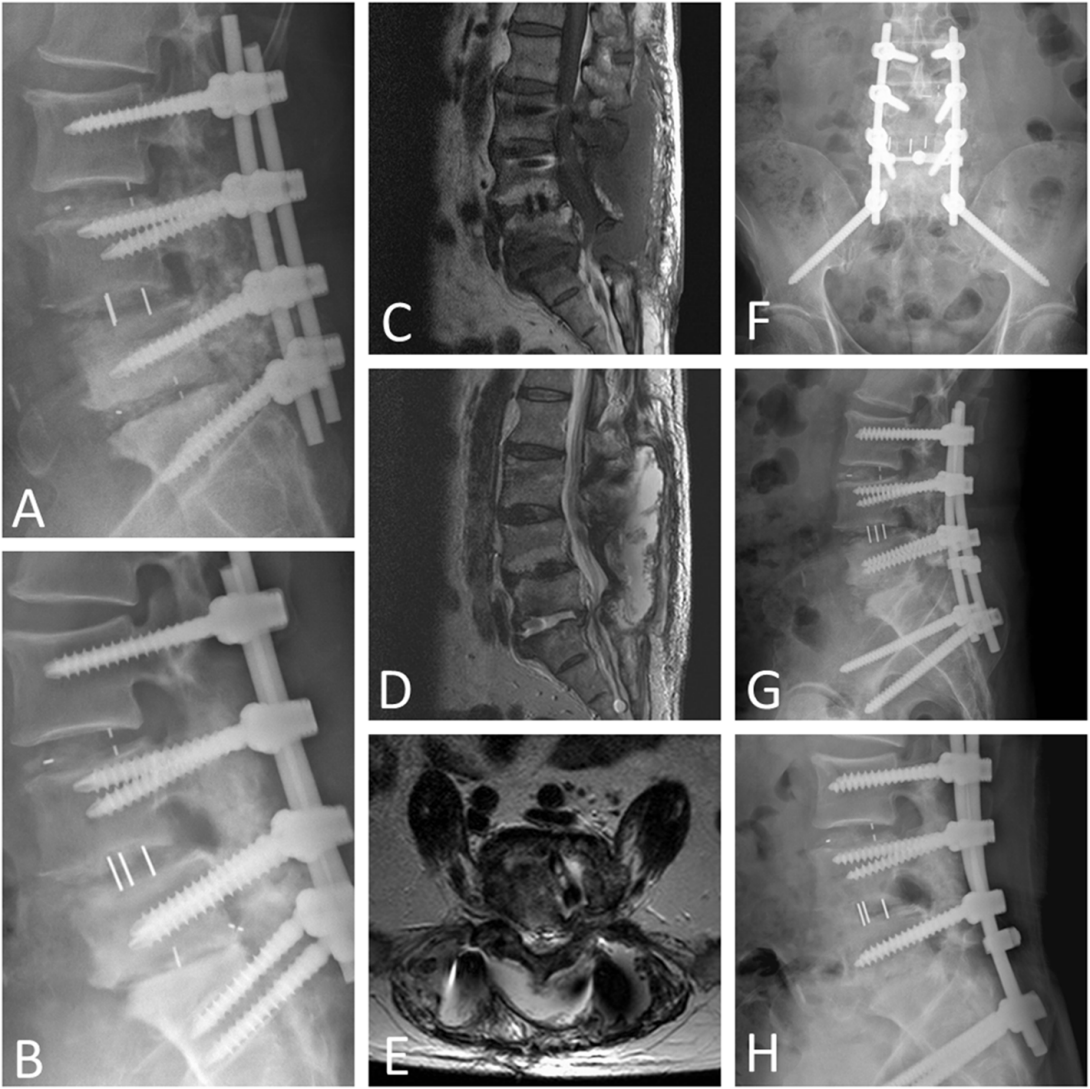
**A** A 57-year-old man underwent L3–S1 fusion with screws and cages. An initial postoperative X-ray showed good locations of the implants. **B** One month after the surgery, a follow-up X-ray showed posterior migration of the L5–S1 cage and suspected concurrent S1 screw loosening. **C**, **D**, **E** Magnetic resonance imaging revealed large amount of fluid accumulation in the L3–S1 subfascial, epidural, posterolateral regions, and L4–L5 disc space. **F**, **G** Posterior-alone surgery with extension of the distal instrumented level, removal of the L5–S1 cage, and L5–S1 TLIDF with allograft were performed. An initial postoperative X-ray showed good alignment. **H** One year later, an X-ray revealed L5–S1 solid interbody union, and the infection was well controlled.

Among the 25 patients with cage infection, the cage was removed via an anterior approach in 12 patients and via a posterior approach in 13 patients. In the anterior group (Fig. 3), the whole procedure was usually composed of anterior cage removal, anterior interbody fusion, and posterior surgery for extension of the instrumented level. Ten out of the 12 patients (83.3%) underwent simultaneous or staged anterior + posterior surgery, and two out of the 12 cases (16.7%) underwent anterior-alone surgery. In the posterior group (Fig. 4), all of the patients (100%) underwent posterior-alone surgery. The comparisons between these two approaches for cage removal are listed in Table 3. No significant difference in mean age, gender, preoperative and 1-year postoperative VAS scores, intraoperative blood loss, or fusion status was observed. However, there was a significant difference in the following items: elapsed time to a diagnosis of cage infection (anterior group: 89 ± 43 days, posterior group: 46 ± 16 days), operative time (anterior group: 274 ± 102 min, posterior group: 163 ± 52 min), and hospital stay/duration of intravenous antibiotics use (anterior group: 35 ± 15 days, posterior group: 23 ± 8 days).

**Table 3:** Comparison between anterior and posterior removal groups

	Anterior (n = 12)	Posterior (n = 13)	***p*** value
Age (years)	65.8 ± 5.2	65.4 ± 11.1	0.917
Elapsed time to a diagnosis (days)	89 ± 43	46 ± 16	0.015*
VAS			
Preoperative 12 months after OP	6.9 ± 1.4 1.33 ± 0.9	7.0 ± 1.4 1.54 ± 1.1	0.885 0.605
Blood loss (ml)	933 ± 576	700 ± 370	0.236
OP time (minutes)	274 ± 102	163 ± 52	0.002*
Hospital stay (days)	35 ± 15	23 ± 8	0.003*
Fusion status			
Solid union Partial union Pseudarthrosis	10 2 0	11 1 1	

*VAS*, visual analogue scale; *OP*, operation

Data presented as mean ± standard deviation, or number of patients

^*^p < 0.05 as statistically significant

## Discussion

**Fig. 5:**
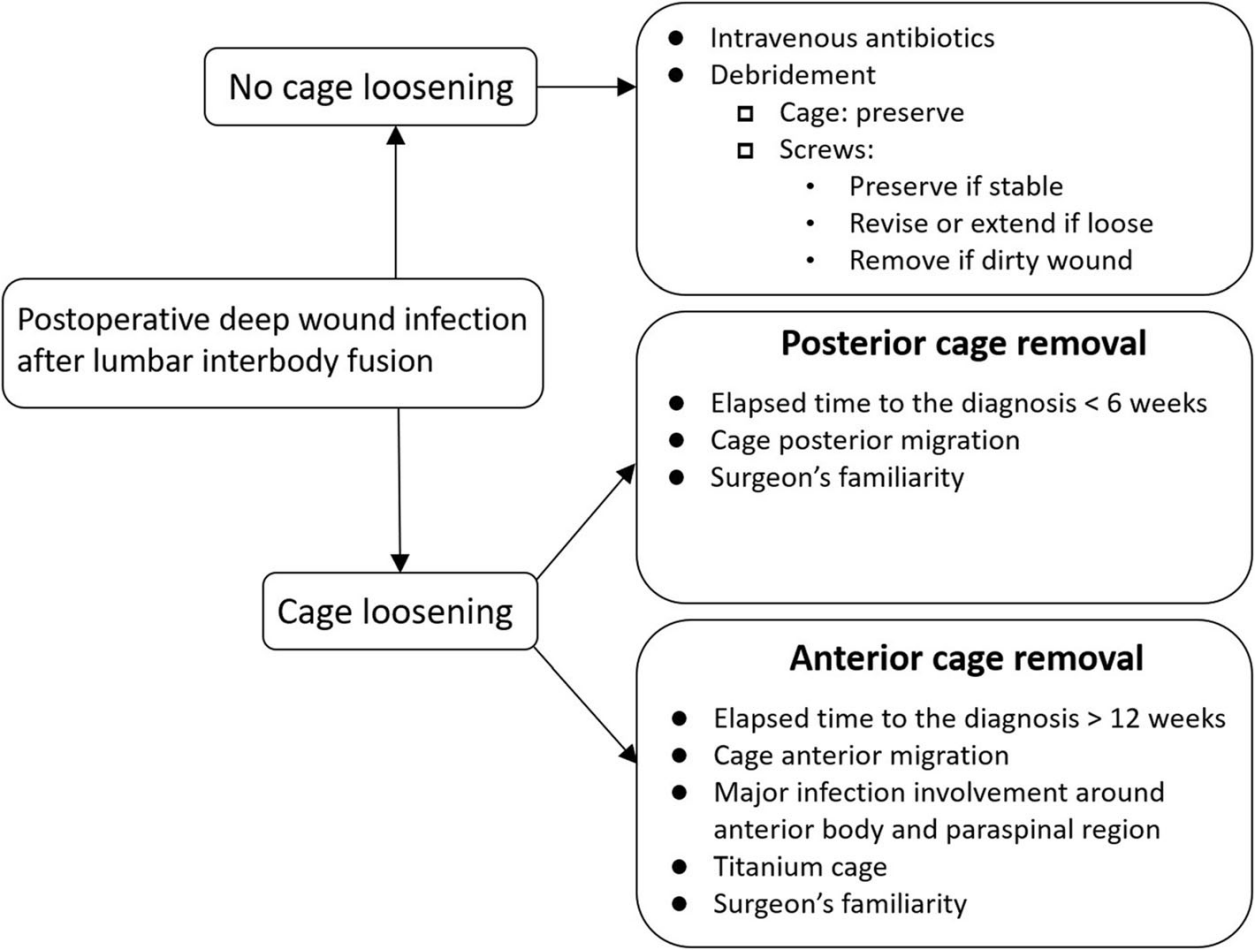
Our treatment algorithms for postoperative deep wound infection after lumbar interbody fusion, which depended on radiographic signs of cage loosening. The indications for anterior removal and posterior removal are listed

In light of the debate regarding the appropriate management of interbody cage infection, this study tried to evaluate the approaches to cage removal and the surgical outcomes of postoperative interbody cage infection in the lumbar spine. We found that both anterior and posterior approaches for cage removal, followed by interbody debridement and fusion with bone grafts, were feasible methods and offered promising infection control, neurologic improvement, and fusion status. Posterior approaches had the advantages of less operative time and shorter hospital stays, and allowed for the cage removal, eradication of infection, and application of instrumentation in a one-stage surgery. Anterior approaches for cage removal usually had to be combined with a posterior approach for the extension of the instrumented level due to the high incidence of pedicle screw loosening (84%). In our case series, the mean elapsed time to the diagnosis of cage infection was shorter in the posterior removal group than the anterior removal group. Therefore, we suggest that a posterior approach can be considered first when the elapsed time to the diagnosis of cage infection is less than 6 weeks, and an endoscope-assisted technique can be utilized to facilitate the cage removal. Once difficulty in cage removal is encountered using a posterior approach, the level of instrumented fusion can still be extended in the posterior surgery and followed by anterior removal. In our opinion, an anterior approach for removal can be considered first based on the following factors: elapsed time to the diagnosis of cage infection of more than 12 weeks (due to expected increased adhesion around the previous TLIF/PLIF entrance), anterior cage migration/dislodgement, prominent infected involvement around anterior body and paraspinal region, the use of a titanium cage (due to a high coefficient of friction), and the surgeon’s familiarity (Fig. 5). If the index surgery was posterior fusion, either anterior or posterior approach was performed to remove the infected case depending on the elapsed time to the diagnosis of cage infection, cage migration area, prominent infected involvement region, and cage material. On the other hand, if the index surgery was anterior fusion, only anterior approach was used to remove infected cage because of larger cage size and different cage axis.

There were several approaches reported in the literature for reaching a loosened cage. Anterior or posterior approaches were initially introduced to pull out infected cages. Afterwards, different methods with specific instruments, such as oblique lumbar interbody fusion (OLIF) and extreme lateral interbody fusion (XLIF), were attempted.

Anterior approaches for cage removal were generally performed because significant scar tissue may be encountered during a second posterior surgery, especially in cases with a long interval between the surgeries [11]. A traditional anterior approach can provide a clear surgical field during cage removal and allow the preparation of endplates for the fusion. Talia et al. also reported that anterior approaches have the advantage of avoiding dissecting the paraspinal muscles, resulting in less postoperative pain and shorter inpatient stays [12]. However, the overall complication rate for anterior approaches was still 14.1%, and the most common complications were venous injury (3.2%) [13]. Vascular injury in revision surgery poses particularly high risks of contributing to retroperitoneal fibrosis formed by the primary approach [14]. A past study reported a 57% vascular injury rate with 89% and 40% complication rates at L4–L5 and L5–S1, respectively [15]. Other than vascular injury, if the hypogastric plexus is injured during an anterior approach, it has been reported to result in retrograde ejaculation in up to 45% of men. Incisional hernia, anterior abdominal muscle atony, and visceral complications such as bowel perforation may also occur in any abdominal surgery [12]. In our series, anterior approaches for cage removal had another shortcoming, which was that their combination with a posterior approach was required for the extension of instrumented level due to the high incidence of concurrent pedicle screw loosening. Such combined anterior and posterior surgeries had significantly longer operative times and resulted in longer hospital stays compared with one-stage posterior removal surgeries.

Although posterior approaches avoid large vessels and visceral injury relative to anterior approaches, they are technically demanding operations. During a second posterior approach, injury to the paravertebral musculature is inevitable, which can increase the risks of postoperative bleeding, infection, myofascial pain, and nerve injury by up to 15–30% [16]. Due to the changes in the natural plane and anatomic landmarks resulting from the prior surgery, epidural fibrosis may make the identification of the previous TLIF/PLIF entrance challenging [17]. Because of the postoperative adhesive dura and nerve roots, there is a high probability of nerve root injuries and dural tearing. Cammisa et al. reported an 8.1% incidence of durotomy for revision spinal surgeries and a 1.0–3.0% rate for primary surgeries [18]. Selznick et al. revealed 66.7% and 21.3% rates of cerebrospinal fluid leak in minimal invasive revision of PLIF and TLIF, respectively [17]. Fortunately, no nerve root injury was noted in our series, which may have resulted from proper patient selection with relatively early revision surgery. In addition, we propose herein that the use of an endoscope-assisted removal technique can facilitate cage removal with a posterior approach. The main advantage of this endoscopy-assisted technique is safety. This technique decreased the risks of vascular and nerve root injuries, which may occur during blind clamping, especially in cases of anterior migration and lateral migration of the infected cage.

Another approach through the pathway of XLIF has gained popularity in revision surgery for avoiding the complications of traditional exposures [14]. The incidence of vascular injury and neural injury is lower with this approach than with the anterior and posterior approaches due to XLIF allowing a pathway to the anterior lumbar spine by splitting through the psoas muscle, thus reducing manipulation of the aorta and vena cava [14, 19]. Other approaches have also been applied for cage removal and fusion, such as OLIF, a psoas-preserving access to the revision level via anterior oblique retroperitoneal approach, or laparoscopic surgery to remove migrated cages in the retroperitoneal space [19, 20].

Regarding the optimal management of postoperative infection, a review of the literature revealed that the elapsed time of infection, the location and severity of infection focus, and the stability of the implant/interbody cage could determine the selection of treatment. For patients suffering from postoperative deep wound infection without radiographic evidence of implant/cage loosening after spinal interbody fusion, intravenous antibiotics plus surgical debridement with implant and cage preservation usually enabled adequate infection control. Pappou et al. conducted a retrospective study of 14 patients with early (presenting at a mean of 18 days) postoperative deep wound infection without implant/cage loosening after ALIF or TLIF [21]. After early debridement and antibiotics use, only one patient with a history of multiple previous spine surgeries had a recurrence of osteomyelitis and needed the removal of the interbody cage with further reconstruction. Similar studies, reported by Lee et al. [6], Mirovsky et al. [8], and Pull ter Gunne et al. [22] revealed high success rates of infection control and improvement of functional outcomes by early debridement with the retention of the implant/cage if there was no evidence of implant/cage instability. Lee et al. [6] found that an elapsed time to a diagnosis of infection longer than 3 months was the independent risk factor for removal of the implant/cage.

If an infection is uncontrollable with sensitive antibiotics and repeated debridement, or if there is any radiographic evidence showing adhesion of the infection to the interbody cage, cage migration, or cage loosening, it is generally accepted that removal and reimplantation of the cage is necessary [6, 8, 23]. Carmouche et al. [23] reported that a patient with cage loosening and purulence surrounding the cage under MRI can be treated by removal of the cage and posterior implants and the performance of posterolateral arthrodesis. Mirovsky et al. [8] found that infected loose PLIF cages could successfully be repositioned or replaced with new bigger cages. However, based on the suspicion of infection recurrence with cage reimplantation and our experience with TLIDF with bone grafts [24], we preferred to use autologous iliac bone grafts or allografts for interbody fusion instead of cage reimplantation. Whether a one-stage or two-stage surgery is used is also an important issue. Immediate implant removal with debridement and later revision surgery after infection control have previously been recommended [25]. In our study, we showed satisfactory outcomes with one-stage surgery with either anterior and posterior surgery or posterior-alone surgery.

Our study had some limitations. Due to the retrospective design of the study, some important clinical characteristics may not have been recorded, which may have introduced unrecognized bias. The surgical options used were not selected randomly, and the surgical team was not unified. The time interval between operations was not the only relevant factor in the decision-making of surgical approach due to the relatively small sample size. In addition, although the mean of the follow-up period in this study was 15.7 months, the shortest follow-up period lasted about 12 months after the surgery. Another limitation was that we used only the recurrence rate, VAS scores, neurologic status, and fusion status for the evaluation of clinical outcomes, while functional scores were not used. Despite these limitations, this study presents the largest series thus far of cases of cage infection with the characteristic of radiographic cage loosening, and provides valuable information regarding the proper management of cage infections.

## Conclusion

For the cases investigated in this study, the removal of a cage was indicated if the impression of a cage infection was indicated preoperatively or intraoperatively. Both anterior and posterior approaches for cage removal, followed by interbody debridement and fusion with bone grafts, were found to be feasible methods that offered promising results. A posterior-alone approach can be considered first when the elapsed time to the diagnosis of cage infection is less than 6 weeks and posterior cage migration has occurred. An endoscope-assisted technique was found to have facilitated cage removal and offered enhanced safety during posterior procedures. An anterior approach is suggested when the elapsed time to the diagnosis of cage infection is more than 12 weeks, anterior cage migration has occurred, there is prominent infected involvement around the anterior body and paraspinal region, or a titanium cage was used (due to the high coefficient of friction). In this series, however, an anterior approach for cage removal usually had to be combined with a posterior approach for extension of the instrumented level due to the high incidence of concurrent pedicle screw loosening.

## Data Availability

All data generated or analyzed during this study are included in this published article.
